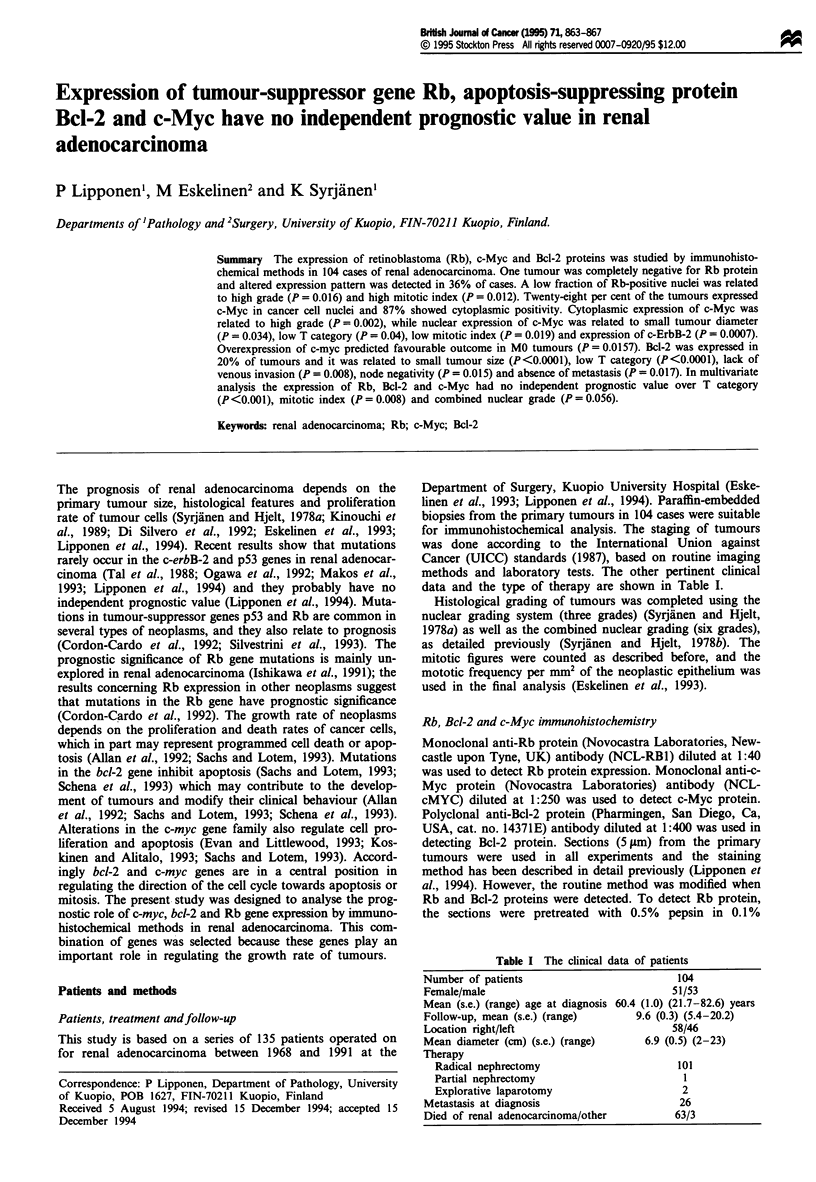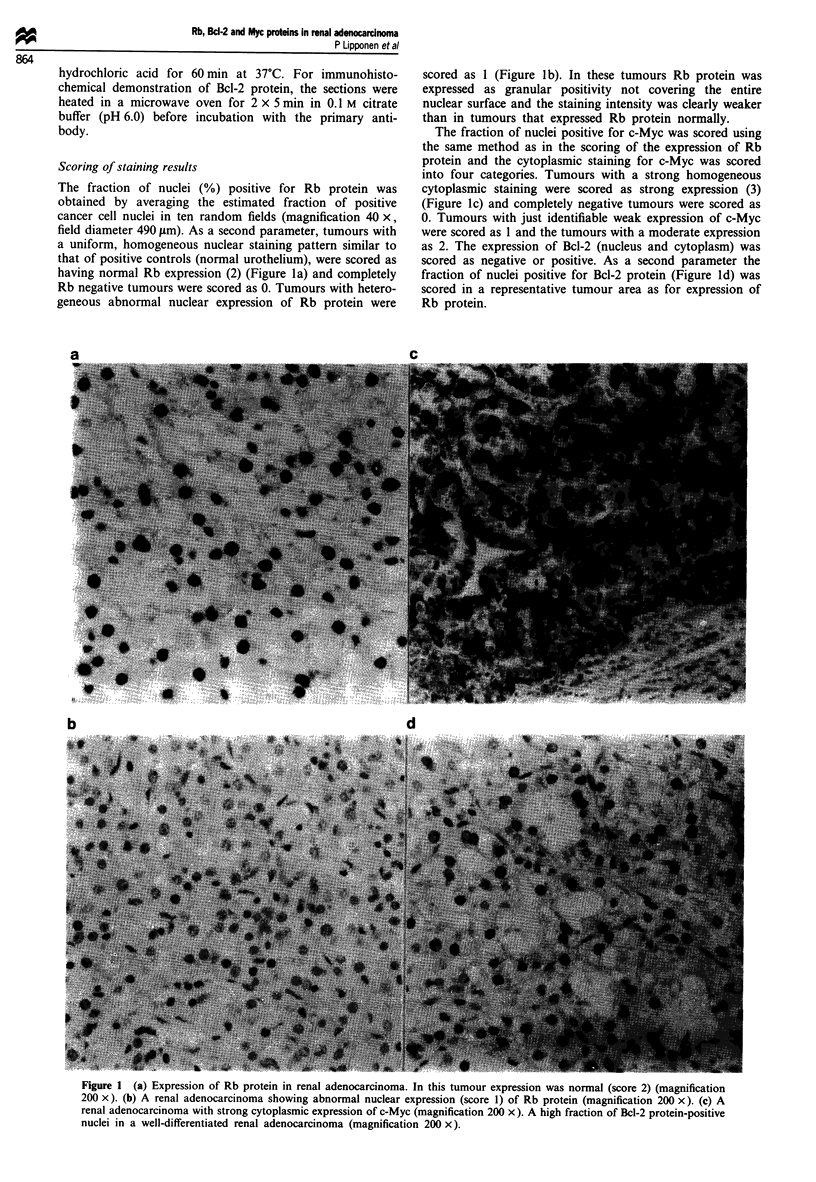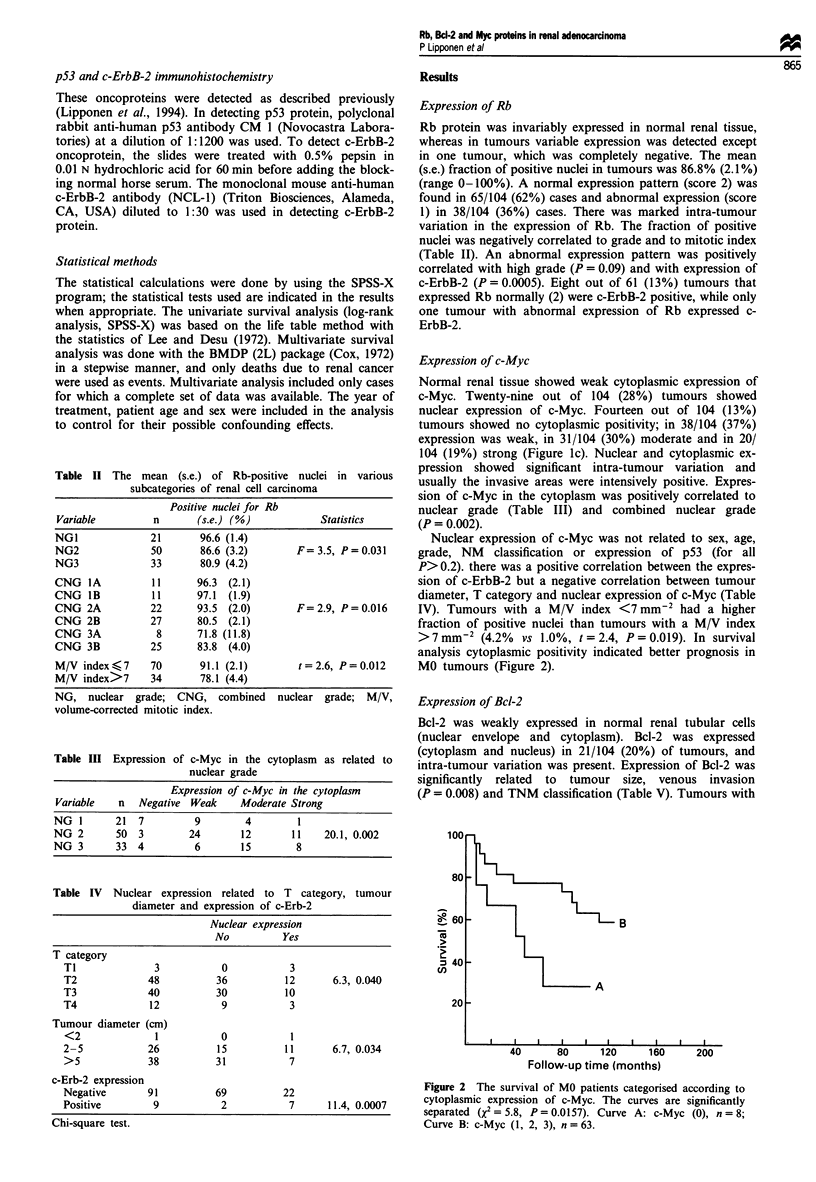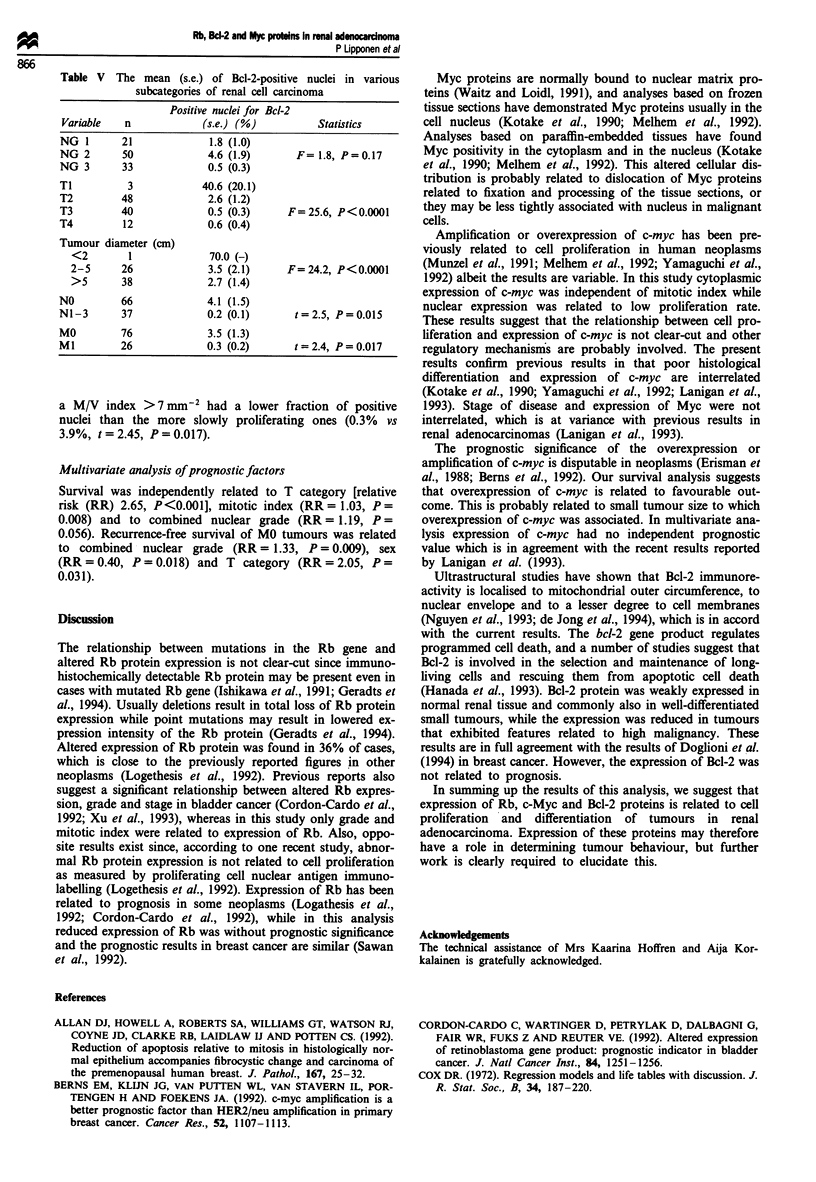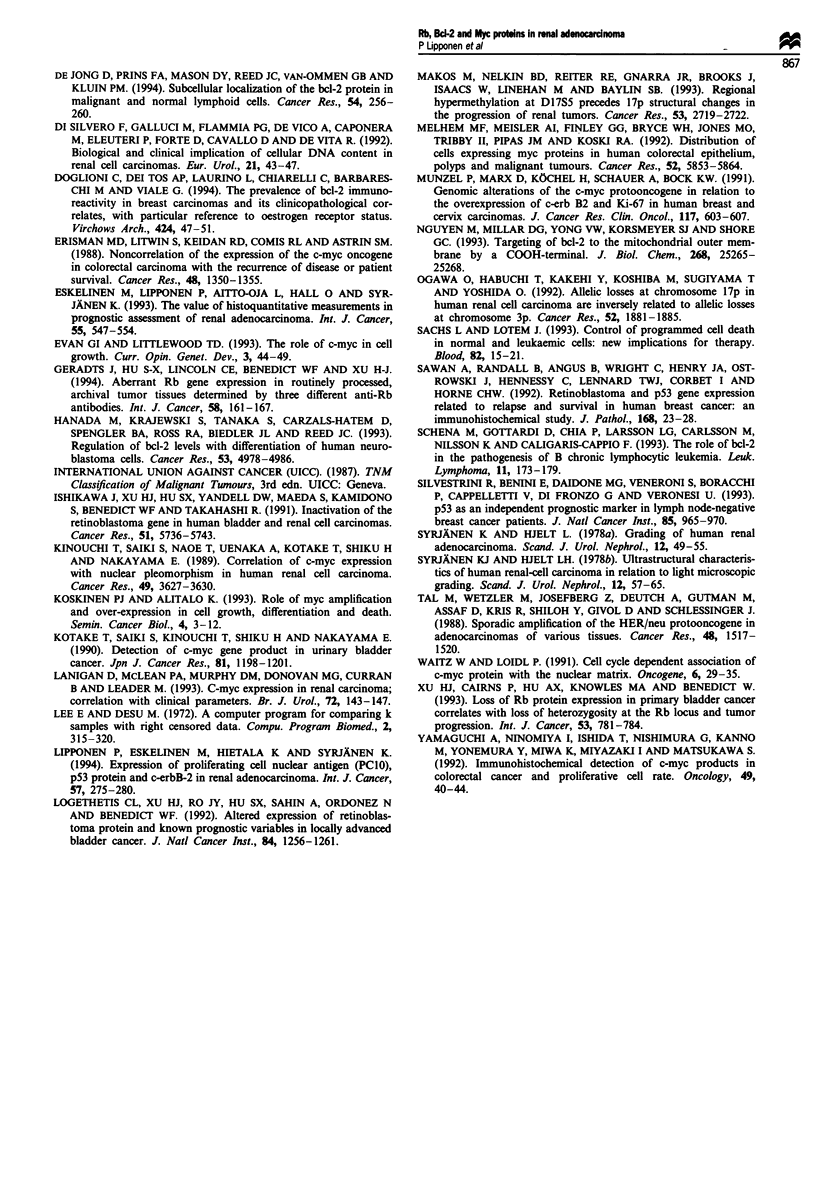# Expression of tumour-suppressor gene Rb, apoptosis-suppressing protein Bcl-2 and c-Myc have no independent prognostic value in renal adenocarcinoma.

**DOI:** 10.1038/bjc.1995.166

**Published:** 1995-04

**Authors:** P. Lipponen, M. Eskelinen, K. Syrjänen

**Affiliations:** Department of Pathology, University of Kuopio, Finland.

## Abstract

**Images:**


British Journal of Cancer (1995) 71, 863-867
© 1995 Stockton Press All rights reserved 0007-0920/95 $12.00

### Expression of tumour-suppressor gene Rb, apoptosis-suppressing protein Bcl-2 and c-Myc have no independent prognostic value in renal adenocarcinoma

P Lipponen[1], M Eskelinen[2] and K Syrjänen[1]

*Departments of [1]Pathology and [2]Surgery, University of Kuopio, FIN-70211 Kuopio, Finland.*

**Summary** The expression of retinoblastoma (Rb), c-Myc and Bcl-2 proteins was studied by immunohistochemical methods in 104 cases of renal adenocarcinoma. One tumour was completely negative for Rb protein and altered expression pattern was detected in 36% of cases. A low fraction of Rb-positive nuclei was related to high grade ($P = 0.016$) and high mitotic index ($P = 0.012$). Twenty-eight per cent of the tumours expressed c-Myc in cancer cell nuclei and 87% showed cytoplasmic positivity. Cytoplasmic expression of c-Myc was related to high grade ($P = 0.002$), while nuclear expression of c-Myc was related to small tumour diameter ($P = 0.034$), low T category ($P = 0.04$), low mitotic index ($P = 0.019$) and expression of c-ErbB-2 ($P = 0.0007$). Overexpression of c-myc predicted favourable outcome in M0 tumours ($P = 0.0157$). Bcl-2 was expressed in 20% of tumours and it was related to small tumour size ($P<0.0001$), low T category ($P<0.0001$), lack of venous invasion ($P = 0.008$), node negativity ($P = 0.015$) and absence of metastasis ($P = 0.017$). In multivariate analysis the expression of Rb, Bcl-2 and c-Myc had no independent prognostic value over T category ($P<0.001$), mitotic index ($P = 0.008$) and combined nuclear grade ($P = 0.056$).

**Keywords:** renal adenocarcinoma; Rb; c-Myc; Bcl-2

The prognosis of renal adenocarcinoma depends on the primary tumour size, histological features and proliferation rate of tumour cells (Syrjänen and Hjelt, 1978*a*; Kinouchi *et al.*, 1989; Di Silvero *et al.*, 1992; Eskelinen *et al.*, 1993; Lipponen *et al.*, 1994). Recent results show that mutations rarely occur in the c-*erb*B-2 and p53 genes in renal adenocarcinoma (Tal *et al.*, 1988; Ogawa *et al.*, 1992; Makos *et al.*, 1993; Lipponen *et al.*, 1994) and they probably have no independent prognostic value (Lipponen *et al.*, 1994). Mutations in tumour-suppressor genes p53 and Rb are common in several types of neoplasms, and they also relate to prognosis (Cordon-Cardo *et al.*, 1992; Silvestrini *et al.*, 1993). The prognostic significance of Rb gene mutations is mainly unexplored in renal adenocarcinoma (Ishikawa *et al.*, 1991); the results concerning Rb expression in other neoplasms suggest that mutations in the Rb gene have prognostic significance (Cordon-Cardo *et al.*, 1992). The growth rate of neoplasms depends on the proliferation and death rates of cancer cells, which in part may represent programmed cell death or apoptosis (Allan *et al.*, 1992; Sachs and Lotem, 1993). Mutations in the *bcl*-2 gene inhibit apoptosis (Sachs and Lotem, 1993; Schena *et al.*, 1993) which may contribute to the development of tumours and modify their clinical behaviour (Allan *et al.*, 1992; Sachs and Lotem, 1993; Schena *et al.*, 1993). Alterations in the c-*myc* gene family also regulate cell proliferation and apoptosis (Evan and Littlewood, 1993; Koskinen and Alitalo, 1993; Sachs and Lotem, 1993). Accordingly *bcl*-2 and c-*myc* genes are in a central position in regulating the direction of the cell cycle towards apoptosis or mitosis. The present study was designed to analyse the prognostic role of c-*myc*, *bcl*-2 and Rb gene expression by immunohistochemical methods in renal adenocarcinoma. This combination of genes was selected because these genes play an important role in regulating the growth rate of tumours.

### Patients and methods

### *Patients, treatment and follow-up*

This study is based on a series of 135 patients operated on for renal adenocarcinoma between 1968 and 1991 at the Department of Surgery, Kuopio University Hospital (Eskelinen *et al.*, 1993; Lipponen *et al.*, 1994). Paraffin-embedded biopsies from the primary tumours in 104 cases were suitable for immunohistochemical analysis. The staging of tumours was done according to the International Union against Cancer (UICC) standards (1987), based on routine imaging methods and laboratory tests. The other pertinent clinical data and the type of therapy are shown in Table I.

Histological grading of tumours was completed using the nuclear grading system (three grades) (Syrjänen and Hjelt, 1978*a*) as well as the combined nuclear grading (six grades), as detailed previously (Syrjänen and Hjelt, 1978*b*). The mitotic figures were counted as described before, and the mototic frequency per $mm^2$ of the neoplastic epithelium was used in the final analysis (Eskelinen *et al.*, 1993).

### *Rb, Bcl-2 and c-Myc immunohistochemistry*

Monoclonal anti-Rb protein (Novocastra Laboratories, Newcastle upon Tyne, UK) antibody (NCL-RB1) diluted at 1:40 was used to detect Rb protein expression. Monoclonal anti-c-Myc protein (Novocastra Laboratories) antibody (NCL-cMYC) diluted at 1:250 was used to detect c-Myc protein. Polyclonal anti-Bcl-2 protein (Pharmingen, San Diego, Ca, USA, cat. no. 14371E) antibody diluted at 1:400 was used in detecting Bcl-2 protein. Sections (5 μm) from the primary tumours were used in all experiments and the staining method has been described in detail previously (Lipponen *et al.*, 1994). However, the routine method was modified when Rb and Bcl-2 proteins were detected. To detect Rb protein, the sections were pretreated with 0.5% pepsin in 0.1%

**Table I** The clinical data of patients

| | |
|---|---|
| Number of patients | 104 |
| Female/male | 51/53 |
| Mean (s.e.) (range) age at diagnosis | 60.4 (1.0) (21.7-82.6) years |
| Follow-up, mean (s.e.) (range) | 9.6 (0.3) (5.4-20.2) |
| Location right/left | 58/46 |
| Mean diameter (cm) (s.e.) (range) | 6.9 (0.5) (2-23) |
| Therapy | |
| Radical nephrectomy | 101 |
| Partial nephrectomy | 1 |
| Explorative laparotomy | 2 |
| Metastasis at diagnosis | 26 |
| Died of renal adenocarcinoma/other | 63/3 |

Correspondence: P Lipponen, Department of Pathology, University of Kuopio, POB 1627, FIN-70211 Kuopio, Finland
Received 5 August 1994; revised 15 December 1994; accepted 15 December 1994

hydrochloric acid for 60 min at 37°C. For immunohistochemical demonstration of Bcl-2 protein, the sections were heated in a microwave oven for 2 × 5 min in 0.1 M citrate buffer (pH 6.0) before incubation with the primary antibody.

*Scoring of staining results*

The fraction of nuclei (%) positive for Rb protein was obtained by averaging the estimated fraction of positive cancer cell nuclei in ten random fields (magnification 40 ×, field diameter 490 μm). As a second parameter, tumours with a uniform, homogeneous nuclear staining pattern similar to that of positive controls (normal urothelium), were scored as having normal Rb expression (2) (Figure 1a) and completely Rb negative tumours were scored as 0. Tumours with heterogeneous abnormal nuclear expression of Rb protein were scored as 1 (Figure 1b). In these tumours Rb protein was expressed as granular positivity not covering the entire nuclear surface and the staining intensity was clearly weaker than in tumours that expressed Rb protein normally.

The fraction of nuclei positive for c-Myc was scored using the same method as in the scoring of the expression of Rb protein and the cytoplasmic staining for c-Myc was scored into four categories. Tumours with a strong homogeneous cytoplasmic staining were scored as strong expression (3) (Figure 1c) and completely negative tumours were scored as 0. Tumours with just identifiable weak expression of c-Myc were scored as 1 and the tumours with a moderate expression as 2. The expression of Bcl-2 (nucleus and cytoplasm) was scored as negative or positive. As a second parameter the fraction of nuclei positive for Bcl-2 protein (Figure 1d) was scored in a representative tumour area as for expression of Rb protein.

**Figure 1** (**a**) Expression of Rb protein in renal adenocarcinoma. In this tumour expression was normal (score 2) (magnification 200 ×). (**b**) A renal adenocarcinoma showing abnormal nuclear expression (score 1) of Rb protein (magnification 200 ×). (**c**) A renal adenocarcinoma with strong cytoplasmic expression of c-Myc (magnification 200 ×). A high fraction of Bcl-2 protein-positive nuclei in a well-differentiated renal adenocarcinoma (magnification 200 ×).

### *p53 and c-ErbB-2 immunohistochemistry*

These oncoproteins were detected as described previously (Lipponen *et al.*, 1994). In detecting p53 protein, polyclonal rabbit anti-human p53 antibody CM 1 (Novocastra Laboratories) at a dilution of 1:1200 was used. To detect c-ErbB-2 oncoprotein, the slides were treated with 0.5% pepsin in 0.01 N hydrochloric acid for 60 min before adding the blocking normal horse serum. The monoclonal mouse anti-human c-ErbB-2 antibody (NCL-1) (Triton Biosciences, Alameda, CA, USA) diluted to 1:30 was used in detecting c-ErbB-2 protein.

### *Statistical methods*

The statistical calculations were done by using the SPSS-X program; the statistical tests used are indicated in the results when appropriate. The univariate survival analysis (log-rank analysis, SPSS-X) was based on the life table method with the statistics of Lee and Desu (1972). Multivariate survival analysis was done with the BMDP (2L) package (Cox, 1972) in a stepwise manner, and only deaths due to renal cancer were used as events. Multivariate analysis included only cases for which a complete set of data was available. The year of treatment, patient age and sex were included in the analysis to control for their possible confounding effects.

**Table II** The mean (s.e.) of Rb-positive nuclei in various subcategories of renal cell carcinoma

| *Variable* | n | *Positive nuclei for Rb (s.e.) (%)* | *Statistics* |
|---|---|---|---|
| NG1 | 21 | 96.6 (1.4) | |
| NG2 | 50 | 86.6 (3.2) | $F = 3.5$, $P = 0.031$ |
| NG3 | 33 | 80.9 (4.2) | |
| CNG 1A | 11 | 96.3 (2.1) | |
| CNG 1B | 11 | 97.1 (1.9) | |
| CNG 2A | 22 | 93.5 (2.0) | $F = 2.9$, $P = 0.016$ |
| CNG 2B | 27 | 80.5 (2.1) | |
| CNG 3A | 8 | 71.8 (11.8) | |
| CNG 3B | 25 | 83.8 (4.0) | |
| M/V index≤7 | 70 | 91.1 (2.1) | $t = 2.6$, $P = 0.012$ |
| M/V index>7 | 34 | 78.1 (4.4) | |

NG, nuclear grade; CNG, combined nuclear grade; M/V, volume-corrected mitotic index.

**Table III** Expression of c-Myc in the cytoplasm as related to nuclear grade

| *Variable* | n | *Negative* | *Weak* | *Moderate* | *Strong* | |
|---|---|---|---|---|---|---|
| NG 1 | 21 | 7 | 9 | 4 | 1 | |
| NG 2 | 50 | 3 | 24 | 12 | 11 | 20.1, 0.002 |
| NG 3 | 33 | 4 | 6 | 15 | 8 | |

(Column group heading: *Expression of c-Myc in the cytoplasm*)

**Table IV** Nuclear expression related to T category, tumour diameter and expression of c-Erb-2

| | | *Nuclear expression No* | *Yes* | |
|---|---|---|---|---|
| T category | | | | |
| T1 | 3 | 0 | 3 | |
| T2 | 48 | 36 | 12 | 6.3, 0.040 |
| T3 | 40 | 30 | 10 | |
| T4 | 12 | 9 | 3 | |
| Tumour diameter (cm) | | | | |
| <2 | 1 | 0 | 1 | |
| 2–5 | 26 | 15 | 11 | 6.7, 0.034 |
| >5 | 38 | 31 | 7 | |
| c-Erb-2 expression | | | | |
| Negative | 91 | 69 | 22 | |
| Positive | 9 | 2 | 7 | 11.4, 0.0007 |

Chi-square test.

### Results

### *Expression of Rb*

Rb protein was invariably expressed in normal renal tissue, whereas in tumours variable expression was detected except in one tumour, which was completely negative. The mean (s.e.) fraction of positive nuclei in tumours was 86.8% (2.1%) (range 0–100%). A normal expression pattern (score 2) was found in 65/104 (62%) cases and abnormal expression (score 1) in 38/104 (36%) cases. There was marked intra-tumour variation in the expression of Rb. The fraction of positive nuclei was negatively correlated to grade and to mitotic index (Table II). An abnormal expression pattern was positively correlated with high grade ($P = 0.09$) and with expression of c-ErbB-2 ($P = 0.0005$). Eight out of 61 (13%) tumours that expressed Rb normally (2) were c-ErbB-2 positive, while only one tumour with abnormal expression of Rb expressed c-ErbB-2.

### *Expression of c-Myc*

Normal renal tissue showed weak cytoplasmic expression of c-Myc. Twenty-nine out of 104 (28%) tumours showed nuclear expression of c-Myc. Fourteen out of 104 (13%) tumours showed no cytoplasmic positivity; in 38/104 (37%) expression was weak, in 31/104 (30%) moderate and in 20/104 (19%) strong (Figure 1c). Nuclear and cytoplasmic expression showed significant intra-tumour variation and usually the invasive areas were intensively positive. Expression of c-Myc in the cytoplasm was positively correlated to nuclear grade (Table III) and combined nuclear grade ($P = 0.002$).

Nuclear expression of c-Myc was not related to sex, age, grade, NM classification or expression of p53 (for all $P > 0.2$). there was a positive correlation between the expression of c-ErbB-2 but a negative correlation between tumour diameter, T category and nuclear expression of c-Myc (Table IV). Tumours with a M/V index $< 7\ mm^{-2}$ had a higher fraction of positive nuclei than tumours with a M/V index $> 7\ mm^{-2}$ (4.2% *vs* 1.0%, $t = 2.4$, $P = 0.019$). In survival analysis cytoplasmic positivity indicated better prognosis in M0 tumours (Figure 2).

### *Expression of Bcl-2*

Bcl-2 was weakly expressed in normal renal tubular cells (nuclear envelope and cytoplasm). Bcl-2 was expressed (cytoplasm and nucleus) in 21/104 (20%) of tumours, and intra-tumour variation was present. Expression of Bcl-2 was significantly related to tumour size, venous invasion ($P = 0.008$) and TNM classification (Table V). Tumours with

**Figure 2** The survival of M0 patients categorised according to cytoplasmic expression of c-Myc. The curves are significantly separated ($\chi^2 = 5.8$, $P = 0.0157$). Curve A: c-Myc (0), $n = 8$; Curve B: c-Myc (1, 2, 3), $n = 63$.

Table V The mean (s.e.) of Bcl-2-positive nuclei in various subcategories of renal cell carcinoma

| Variable | n | Positive nuclei for Bcl-2 (s.e.) (%) | Statistics |
|---|---|---|---|
| NG 1 | 21 | 1.8 (1.0) | |
| NG 2 | 50 | 4.6 (1.9) | $F=1.8$, $P=0.17$ |
| NG 3 | 33 | 0.5 (0.3) | |
| T1 | 3 | 40.6 (20.1) | |
| T2 | 48 | 2.6 (1.2) | |
| T3 | 40 | 0.5 (0.3) | $F=25.6$, $P<0.0001$ |
| T4 | 12 | 0.6 (0.4) | |
| Tumour diameter (cm) | | | |
| <2 | 1 | 70.0 (–) | |
| 2–5 | 26 | 3.5 (2.1) | $F=24.2$, $P<0.0001$ |
| >5 | 38 | 2.7 (1.4) | |
| N0 | 66 | 4.1 (1.5) | |
| N1–3 | 37 | 0.2 (0.1) | $t=2.5$, $P=0.015$ |
| M0 | 76 | 3.5 (1.3) | |
| M1 | 26 | 0.3 (0.2) | $t=2.4$, $P=0.017$ |

a M/V index $>7\ mm^{-2}$ had a lower fraction of positive nuclei than the more slowly proliferating ones (0.3% *vs* 3.9%, $t=2.45$, $P=0.017$).

*Multivariate analysis of prognostic factors*

Survival was independently related to T category [relative risk (RR) 2.65, $P<0.001$], mitotic index (RR = 1.03, $P=0.008$) and to combined nuclear grade (RR = 1.19, $P=0.056$). Recurrence-free survival of M0 tumours was related to combined nuclear grade (RR = 1.33, $P=0.009$), sex (RR = 0.40, $P=0.018$) and T category (RR = 2.05, $P=0.031$).

### Discussion

The relationship between mutations in the Rb gene and altered Rb protein expression is not clear-cut since immunohistochemically detectable Rb protein may be present even in cases with mutated Rb gene (Ishikawa *et al.*, 1991; Geradts *et al.*, 1994). Usually deletions result in total loss of Rb protein expression while point mutations may result in lowered expression intensity of the Rb protein (Geradts *et al.*, 1994). Altered expression of Rb protein was found in 36% of cases, which is close to the previously reported figures in other neoplasms (Logethesis *et al.*, 1992). Previous reports also suggest a significant relationship between altered Rb expression, grade and stage in bladder cancer (Cordon-Cardo *et al.*, 1992; Xu *et al.*, 1993), whereas in this study only grade and mitotic index were related to expression of Rb. Also, opposite results exist since, according to one recent study, abnormal Rb protein expression is not related to cell proliferation as measured by proliferating cell nuclear antigen immunolabelling (Logethesis *et al.*, 1992). Expression of Rb has been related to prognosis in some neoplasms (Logathesis *et al.*, 1992; Cordon-Cardo *et al.*, 1992), while in this analysis reduced expression of Rb was without prognostic significance and the prognostic results in breast cancer are similar (Sawan *et al.*, 1992).

Myc proteins are normally bound to nuclear matrix proteins (Waitz and Loidl, 1991), and analyses based on frozen tissue sections have demonstrated Myc proteins usually in the cell nucleus (Kotake *et al.*, 1990; Melhem *et al.*, 1992). Analyses based on paraffin-embedded tissues have found Myc positivity in the cytoplasm and in the nucleus (Kotake *et al.*, 1990; Melhem *et al.*, 1992). This altered cellular distribution is probably related to dislocation of Myc proteins related to fixation and processing of the tissue sections, or they may be less tightly associated with nucleus in malignant cells.

Amplification or overexpression of c-*myc* has been previously related to cell proliferation in human neoplasms (Munzel *et al.*, 1991; Melhem *et al.*, 1992; Yamaguchi *et al.*, 1992) albeit the results are variable. In this study cytoplasmic expression of c-*myc* was independent of mitotic index while nuclear expression was related to low proliferation rate. These results suggest that the relationship between cell proliferation and expression of c-*myc* is not clear-cut and other regulatory mechanisms are probably involved. The present results confirm previous results in that poor histological differentiation and expression of c-*myc* are interrelated (Kotake *et al.*, 1990; Yamaguchi *et al.*, 1992; Lanigan *et al.*, 1993). Stage of disease and expression of Myc were not interrelated, which is at variance with previous results in renal adenocarcinomas (Lanigan *et al.*, 1993).

The prognostic significance of the overexpression or amplification of c-*myc* is disputable in neoplasms (Erisman *et al.*, 1988; Berns *et al.*, 1992). Our survival analysis suggests that overexpression of c-*myc* is related to favourable outcome. This is probably related to small tumour size to which overexpression of c-*myc* was associated. In multivariate analysis expression of c-*myc* had no independent prognostic value which is in agreement with the recent results reported by Lanigan *et al.* (1993).

Ultrastructural studies have shown that Bcl-2 immunoreactivity is localised to mitochondrial outer circumference, to nuclear envelope and to a lesser degree to cell membranes (Nguyen *et al.*, 1993; de Jong *et al.*, 1994), which is in accord with the current results. The *bcl*-2 gene product regulates programmed cell death, and a number of studies suggest that Bcl-2 is involved in the selection and maintenance of long-living cells and rescuing them from apoptotic cell death (Hanada *et al.*, 1993). Bcl-2 protein was weakly expressed in normal renal tissue and commonly also in well-differentiated small tumours, while the expression was reduced in tumours that exhibited features related to high malignancy. These results are in full agreement with the results of Doglioni *et al.* (1994) in breast cancer. However, the expression of Bcl-2 was not related to prognosis.

In summing up the results of this analysis, we suggest that expression of Rb, c-Myc and Bcl-2 proteins is related to cell proliferation and differentiation of tumours in renal adenocarcinoma. Expression of these proteins may therefore have a role in determining tumour behaviour, but further work is clearly required to elucidate this.

**Acknowledgements**
The technical assistance of Mrs Kaarina Hoffren and Aija Korkalainen is gratefully acknowledged.

### References

ALLAN DJ, HOWELL A, ROBERTS SA, WILLIAMS GT, WATSON RJ, COYNE JD, CLARKE RB, LAIDLAW IJ AND POTTEN CS. (1992). Reduction of apoptosis relative to mitosis in histologically normal epithelium accompanies fibrocystic change and carcinoma of the premenopausal human breast. *J. Pathol.*, **167**, 25–32.

BERNS EM, KLIJN JG, VAN PUTTEN WL, VAN STAVERN IL, PORTENGEN H AND FOEKENS JA. (1992). c-myc amplification is a better prognostic factor than HER2/neu amplification in primary breast cancer. *Cancer Res.*, **52**, 1107–1113.

CORDON-CARDO C, WARTINGER D, PETRYLAK D, DALBAGNI G, FAIR WR, FUKS Z AND REUTER VE. (1992). Altered expression of retinoblastoma gene product: prognostic indicator in bladder cancer. *J. Natl Cancer Inst.*, **84**, 1251–1256.

COX DR. (1972). Regression models and life tables with discussion. *J. R. Stat. Soc., B*, **34**, 187–220.

DE JONG D, PRINS FA, MASON DY, REED JC, VAN-OMMEN GB AND KLUIN PM. (1994). Subcellular localization of the bcl-2 protein in malignant and normal lymphoid cells. *Cancer Res.*, **54**, 256-260.

DI SILVERO F, GALLUCI M, FLAMMIA PG, DE VICO A, CAPONERA M, ELEUTERI P, FORTE D, CAVALLO D AND DE VITA R. (1992). Biological and clinical implication of cellular DNA content in renal cell carcinomas. *Eur. Urol.*, **21**, 43-47.

DOGLIONI C, DEI TOS AP, LAURINO L, CHIARELLI C, BARBARESCHI M AND VIALE G. (1994). The prevalence of bcl-2 immunoreactivity in breast carcinomas and its clinicopathological correlates, with particular reference to oestrogen receptor status. *Virchows Arch.*, **424**, 47-51.

ERISMAN MD, LITWIN S, KEIDAN RD, COMIS RL AND ASTRIN SM. (1988). Noncorrelation of the expression of the c-myc oncogene in colorectal carcinoma with the recurrence of disease or patient survival. *Cancer Res.*, **48**, 1350-1355.

ESKELINEN M, LIPPONEN P, AITTO-OJA L, HALL O AND SYRJÄNEN K. (1993). The value of histoquantitative measurements in prognostic assessment of renal adenocarcinoma. *Int. J. Cancer*, **55**, 547-554.

EVAN GI AND LITTLEWOOD TD. (1993). The role of c-myc in cell growth. *Curr. Opin. Genet. Dev.*, **3**, 44-49.

GERADTS J, HU S-X, LINCOLN CE, BENEDICT WF AND XU H-J. (1994). Aberrant Rb gene expression in routinely processed, archival tumor tissues determined by three different anti-Rb antibodies. *Int. J. Cancer*, **58**, 161-167.

HANADA M, KRAJEWSKI S, TANAKA S, CARZALS-HATEM D, SPENGLER BA, ROSS RA, BIEDLER JL AND REED JC. (1993). Regulation of bcl-2 levels with differentiation of human neuroblastoma cells. *Cancer Res.*, **53**, 4978-4986.

INTERNATIONAL UNION AGAINST CANCER (UICC). (1987). *TNM Classification of Malignant Tumours*, 3rd edn. UICC: Geneva.

ISHIKAWA J, XU HJ, HU SX, YANDELL DW, MAEDA S, KAMIDONO S, BENEDICT WF AND TAKAHASHI R. (1991). Inactivation of the retinoblastoma gene in human bladder and renal cell carcinomas. *Cancer Res.*, **51**, 5736-5743.

KINOUCHI T, SAIKI S, NAOE T, UENAKA A, KOTAKE T, SHIKU H AND NAKAYAMA E. (1989). Correlation of c-myc expression with nuclear pleomorphism in human renal cell carcinoma. *Cancer Res.*, **49**, 3627-3630.

KOSKINEN PJ AND ALITALO K. (1993). Role of myc amplification and over-expression in cell growth, differentiation and death. *Semin. Cancer Biol.*, **4**, 3-12.

KOTAKE T, SAIKI S, KINOUCHI T, SHIKU H AND NAKAYAMA E. (1990). Detection of c-myc gene product in urinary bladder cancer. *Jpn J. Cancer Res.*, **81**, 1198-1201.

LANIGAN D, McLEAN PA, MURPHY DM, DONOVAN MG, CURRAN B AND LEADER M. (1993). C-myc expression in renal carcinoma; correlation with clinical parameters. *Br. J. Urol.*, **72**, 143-147.

LEE E AND DESU M. (1972). A computer program for comparing k samples with right censored data. *Compu. Program Biomed.*, **2**, 315-320.

LIPPONEN P, ESKELINEN M, HIETALA K AND SYRJÄNEN K. (1994). Expression of proliferating cell nuclear antigen (PC10), p53 protein and c-erbB-2 in renal adenocarcinoma. *Int. J. Cancer*, **57**, 275-280.

LOGETHETIS CL, XU HJ, RO JY, HU SX, SAHIN A, ORDONEZ N AND BENEDICT WF. (1992). Altered expression of retinoblastoma protein and known prognostic variables in locally advanced bladder cancer. *J. Natl Cancer Inst.*, **84**, 1256-1261.

MAKOS M, NELKIN BD, REITER RE, GNARRA JR, BROOKS J, ISAACS W, LINEHAN M AND BAYLIN SB. (1993). Regional hypermethylation at D17S5 precedes 17p structural changes in the progression of renal tumors. *Cancer Res.*, **53**, 2719-2722.

MELHEM MF, MEISLER AI, FINLEY GG, BRYCE WH, JONES MO, TRIBBY II, PIPAS JM AND KOSKI RA. (1992). Distribution of cells expressing myc proteins in human colorectal epithelium, polyps and malignant tumours. *Cancer Res.*, **52**, 5853-5864.

MUNZEL P, MARX D, KÖCHEL H, SCHAUER A, BOCK KW. (1991). Genomic alterations of the c-myc protooncogene in relation to the overexpression of c-erb B2 and Ki-67 in human breast and cervix carcinomas. *J. Cancer Res. Clin. Oncol.*, **117**, 603-607.

NGUYEN M, MILLAR DG, YONG VW, KORSMEYER SJ AND SHORE GC. (1993). Targeting of bcl-2 to the mitochondrial outer membrane by a COOH-terminal. *J. Biol. Chem.*, **268**, 25265-25268.

OGAWA O, HABUCHI T, KAKEHI Y, KOSHIBA M, SUGIYAMA T AND YOSHIDA O. (1992). Allelic losses at chromosome 17p in human renal cell carcinoma are inversely related to allelic losses at chromosome 3p. *Cancer Res.*, **52**, 1881-1885.

SACHS L AND LOTEM J. (1993). Control of programmed cell death in normal and leukaemic cells: new implications for therapy. *Blood*, **82**, 15-21.

SAWAN A, RANDALL B, ANGUS B, WRIGHT C, HENRY JA, OSTROWSKI J, HENNESSY C, LENNARD TWJ, CORBET I AND HORNE CHW. (1992). Retinoblastoma and p53 gene expression related to relapse and survival in human breast cancer: an immunohistochemical study. *J. Pathol.*, **168**, 23-28.

SCHENA M, GOTTARDI D, CHIA P, LARSSON LG, CARLSSON M, NILSSON K AND CALIGARIS-CAPPIO F. (1993). The role of bcl-2 in the pathogenesis of B chronic lymphocytic leukemia. *Leuk. Lymphoma*, **11**, 173-179.

SILVESTRINI R, BENINI E, DAIDONE MG, VENERONI S, BORACCHI P, CAPPELLETTI V, DI FRONZO G AND VERONESI U. (1993). p53 as an independent prognostic marker in lymph node-negative breast cancer patients. *J. Natl Cancer Inst.*, **85**, 965-970.

SYRJÄNEN K AND HJELT L. (1978*a*). Grading of human renal adenocarcinoma. *Scand. J. Urol. Nephrol.*, **12**, 49-55.

SYRJÄNEN KJ AND HJELT LH. (1978*b*). Ultrastructural characteristics of human renal-cell carcinoma in relation to light microscopic grading. *Scand. J. Urol. Nephrol.*, **12**, 57-65.

TAL M, WETZLER M, JOSEFBERG Z, DEUTCH A, GUTMAN M, ASSAF D, KRIS R, SHILOH Y, GIVOL D AND SCHLESSINGER J. (1988). Sporadic amplification of the HER/neu protooncogene in adenocarcinomas of various tissues. *Cancer Res.*, **48**, 1517-1520.

WAITZ W AND LOIDL P. (1991). Cell cycle dependent association of c-myc protein with the nuclear matrix. *Oncogene*, **6**, 29-35.

XU HJ, CAIRNS P, HU AX, KNOWLES MA AND BENEDICT W. (1993). Loss of Rb protein expression in primary bladder cancer correlates with loss of heterozygosity at the Rb locus and tumor progression. *Int. J. Cancer*, **53**, 781-784.

YAMAGUCHI A, NINOMIYA I, ISHIDA T, NISHIMURA G, KANNO M, YONEMURA Y, MIWA K, MIYAZAKI I AND MATSUKAWA S. (1992). Immunohistochemical detection of c-myc products in colorectal cancer and proliferative cell rate. *Oncology*, **49**, 40-44.